# Dataset on The Cultural Dimension of Urban Society Food Consumption in Indonesia

**DOI:** 10.1016/j.dib.2020.105681

**Published:** 2020-05-13

**Authors:** Francisia SSE Seda, Lugina Setyawati, Timoti Tirta, Kevin Nobel

**Affiliations:** Department of Sociology, Universitas Indonesia, Depok, Indonesia

**Keywords:** Cultural dimension of food consumption, Consumerism, Urban society, Social class

## Abstract

This article introduces a dataset that presents the cultural values and practices of food consumption patterns in major urban communities in Indonesian cities. The data illustrate the cultural characteristics of urban residents’ food consumption patterns based on social class categories. Data collection was conducted in five major Indonesian cities through face-to-face interviews with 710 respondents identified using a stratified random sampling technique. The data show that culture has a dominant influence on the pattern of food consumption of urban communities in Indonesia in comparison with economic and health dimensions. Although the value and practice sub-dimensions are conceptually related, the cultural dimension of food consumption patterns in the five urban communities is more dominated by religious value than other cultural practices. Regarding food consumption patterns, urban upper classes are more dominantly influenced by economic dimensions and modern healthy lifestyles than cultural dimensions.

Specification table

**Table:** 

Subject	Sociology, consumerism
Specific subject area	Cultural dimension of food consumption, urban lifestyle, social class
Type of data	Tables, figures
How data were acquired	Field survey
Data format	Raw and descriptive
Parameter for data collection	The questionnaire was developed to measure the cultural dimension of food consumption. A set of indicators are operationalized from the sub-dimensions of cultural values and cultural practice. All items related to the cultural dimension in this dataset are measured using a five-point Likert scale.
	The questionnaire also gathered respondents’ demographic characteristics, household income and expenditure, and the importance of health and price factors in their food consumption patterns
Description of data collection	Data were collected through a survey of 710 respondents from five major cities in Indonesia: Jakarta with 174 samples, Bandung with 150 samples, Surabaya with 118 samples, Makassar with 120 samples, and Denpasar with 148 samples.
	Samples were drawn using a stratified random sampling technique to obtain proportional samples of each social class. The surveys were conducted through face-to-face interviews, with which the research team was assisted by several local enumerators from local universities.
Data source location	Indonesia
Data accessibility	Data included in this article
Related research article	Consumerism Indicators Construction: A Portrait of Household Food Consumption in Surabaya. Global Journal of Human Social Science Vol 14: 7

## Value of the data

• The cultural indicators of food consumption can significantly complement global consumerism literature, which is dominated by analyses of the economic and social dimensions of food consumption.
• In the era of economic globalization, these indicators and data are very important for scholars who are interested in developing an integrative consumerism index, which may be a valid and reliable instrument for research on the cultural characteristics of urban communities in developing countries.
• These data and indicators are also important for the formulation of governmental policies regarding social welfare and the implementation of the UN's Sustainable Development Goals within the scope of the socio-cultural dimension.
• These data and indicators also present important insights for non-governmental organizations to optimize the cultural dimension in poverty alleviation programs.
• A comprehensive understanding of the domestic economy at the micro level can provide input for the government in the formulation of policies to accommodate the economic process of consumerism.

## 1 Data description

**Figure 1:**
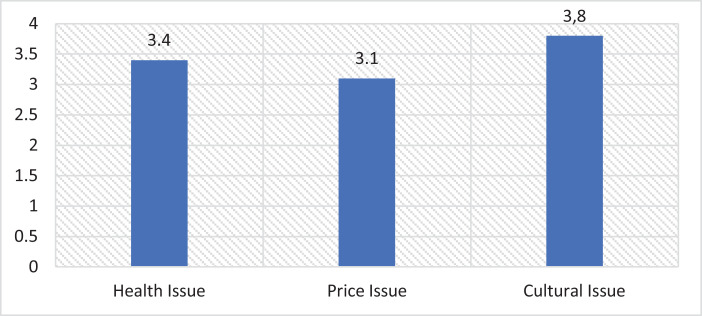
Consideration factors for household food consumption.

**Figure 2:**
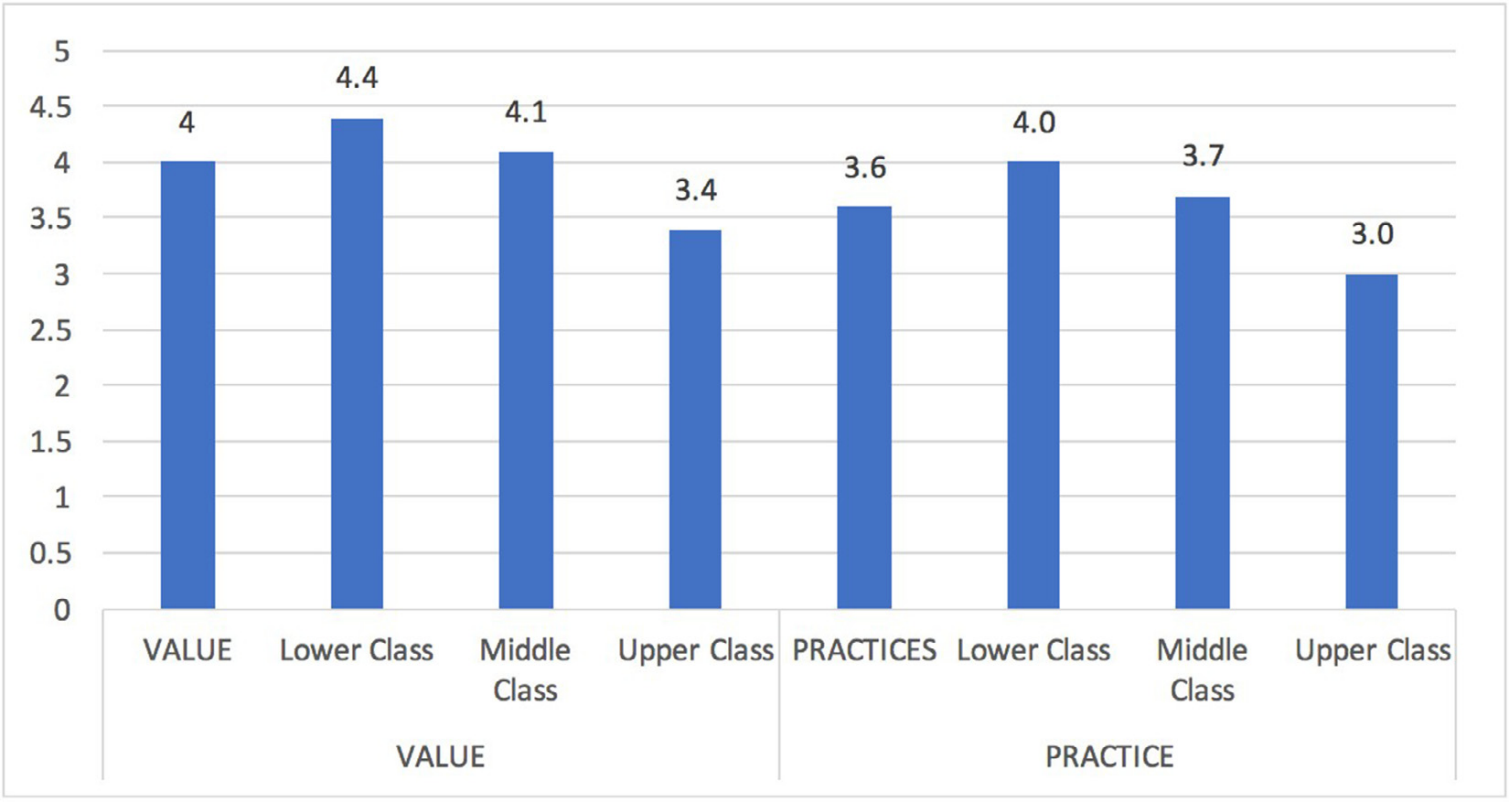
Value and practice sub-dimensions by social class.

The dataset in this article describes the cultural characteristics of urban residents’ food consumption patterns gathered through field surveys conducted in 2015-2016. Table 1 presents a categorization of respondents’ demographic data [1]. In Table 2, the authors categorize respondents by lower, middle, and upper social class households in Indonesia's five major cities. Table 3 presents dimensions, sub-dimensions, and indicators that were used in this survey. Table 4 shows the sample and response rate numbers. Table 5 describes the type of data analysis. Table 6 presents respondents’ incomes and expenditures, while Figure 1 shows the consideration factors of household food consumption. Table 7 categorizes income and expenditure based on the three factors of health, price, and cultural issues. Figure 2 shows the value and practice sub-dimensions by social class. Lastly, Table 8 presents the consideration factors of household food consumption.

**Table 1:** Descriptive Statistics of Demographic Data.

Gender			Highest Education		
	Male (%)	42,1		Primary school (%)	3,4
	Female (%)	57,9		Junior high school (%)	5,2
Average Age (years)	41,3			Senior high school (%)	25,5
Religion				Diploma (%)	22,0
	Islam (%)	60,1		Bachelor's degree (%)	29,9
	Catholic (%)	14,2		Master's and doctoral degree (%)	14,1
	Protestant (%)	15,8	Lived in urban areas (average years)	28,7	
	Hindu (%)	9,3	Occupation		
	Buddhist (%)	0,4		Government employee (%)	28,5
	Other (%)	0,1		Police officer/Military (%)	4,2
Ethnicity				Employee of a private company (%)	28,9
	Java (%)	40,7		Teacher/Lecturer (%)	2,8
	Sunda (%)	13,7		Entrepreneur (%)	27,2
	Minang (%)	8,6		Independent worker (%)	8,5
	Bali (%)	10,3	Social Class		
	Batak (%)	7,2		Lower (%)	34,5
	Madura (%)	8,2		Middle (%)	36,2
	Bugis (%)	3,5		Upper (%)	29,3
	Other (%)	7,9	Average number of household members	6	
City			Province		
	Jakarta (%)	24,5		DKI Jakarta (%)	24,5
	Bandung (%)	21,1		West Java (%)	21,1
	Surabaya (%)	16,6		East Java (%)	16,6
	Makassar (%)	16,9		South Sulawesi (%)	16,9
	Denpasar (%)	20,8		Bali (%)	20,8

**Table 2:** Distribution of Respondent by Social Class and City.

City	Lower Class		Middle Class		Upper Class	
	Freq.	(%)	Freq.	%	Freq.	%
Jakarta	65	37,4	57	32,8	52	29,9
Bandung	50	33,3	57	38,0	43	28,7
Surabaya	40	33,9	43	36,4	35	29,7
Makassar	41	34,2	44	36,7	35	29,2
Denpasar	50	33,8	55	37,2	43	29,1
Total/Average	246	34,5	256	36,2	208	29,3

**Table 3:** Cultural Dimension and Indicators.

Dimension	Sub-Dimension	Indicators	Adapted References
CULTURAL	CULTURAL VALUES	• Religious values/principles regarding types of food	[2]
Subsistent consumption activities associated with food (primary, secondary, and tertiary) of individuals and families affected by a set of cultural elements	Cultural values or principles that serve as a reference in making choices related to food consumption	• Religious values/principles regarding locations for eating • Religious values/principles regarding consumption patterns • Traditional/cultural values regarding types of foods • Value of tradition/customs regarding locations for eating • Traditional/customary values regarding consumption patterns	
	
	CULTURAL PRACTICES	• The practice of buying foods recommended by religious rules	[3], [4], [5], [6], [7]
	Cultural practices related to food consumption are carried out because they refer to cultural principles and values	• The practice of buying recommended types of food according to customary traditions • The practice of choosing to buy food in safe places according to religious rules • Consumption patterns based on religious rules • The practice of consuming certain traditional foods

**Table 4:** Survey Sample and Response Rate.

City	Sample	Response rate	Sub-districts
Jakarta	174	76%	4
Bandung	150	81%	2
Surabaya	118	82%	4
Makassar	120	84%	3
Denpasar	148	87%	2
Total/Average	710	82%	3

**Table 5:** Type of Data Analysis.

Analysis	Aspects	Data	Detail
Univariate	Demographic information	City, Province, Age, Gender, Ethnicity, Religion, Education level, Occupation, Social class, Number of household members	Frequency table, bar chart, mean, %
	Income and expenditure	Household income, household expenditure, Household expenditure for food	Mean
	Consumption aspects	Cultural aspects, Health and price principal of food consumption	Mean
Crosstab analysis	Consumption aspects	Cultural aspects, Health and price principal of food consumption by social class, religion, and ethnicity	Crosstable

**Table 6:** Income and Expenditure Characteristics.

Average of total household income/month (in million IDR)		**6,7**
	Lower Class	3,6
	Middle Class	5,9
	Upper Class	11,2
Frequency of income reception		
	Daily	34,1
	Weekly	36,9
	Monthly	29,0
Average of total household expenditure/month (in million IDR)		**6,4**
	Lower Class	3,4
	Middle Class	5,6
	Upper Class	10,8
Monthly household expenditure For Food (%)		**42,9**
	Lower Class	59,7
	Middle Class	40,4
	Upper Class	26,4

**Table 7:** Income and expenditure characteristics based on three factors.

Health issues		**3.4**
	Lower Class	2.8
	Middle Class	3.4
	Upper Class	3.9
Price issues		**3.1**
	Lower Class	3.5
	Middle Class	3.2
	Upper Class	2.7
Cultural issues		**3.8**
	Lower Class	4.2
	Middle Class	3.9
	Upper Class	3.2

**Table 8:** Mean data of Value and Practice indicators.

Sub-dimension	Indicators	Mean
VALUE	Religious values/principles regarding types of food	4.4
	Religious values/principles regarding locations for eating	4.1
	Religious values/principles regarding consumption patterns	4.2
	Traditional/cultural values regarding types of food	4
	Value of tradition/customs regarding locations for eating	3.6
	Traditional/customary values regarding consumption patterns	3.6
PRACTICE	The practice of buying foods recommended by religious rules	3.9
	The practice of buying recommended types of food according to customary traditions	3.4
	The practice of choosing to buy food in safe places according to religious rules	3.7
	Consumption patterns based on religious rules	3.4
	The practice of consuming certain traditional foods	3.5

The majority of the respondents were female (58%). Considering religious affiliation, the majority of respondents were Muslim (60.1%) which reflects the majority religion in Indonesia. The majority of respondents came from the Javanese ethnic group (40.7%) who are spread across the five cities that were surveyed, while other ethnicities are the majority in certain cities, such as the Sundanese in Bandung, the Balinese in Denpasar, and the Bugis in Makassar. The average age of respondents was 41.3 years and on average, they had lived in the city or another urban area for around 28.7 years.

The majority of respondents had a bachelor's degree (29.9%), high school education (25.5%), a diploma (22%), and a postgraduate degree – master's and doctoral level of education (14.1%). Respondents’ level of education may have had implications on their food consumption choices, depicting dynamics of economic rationality in combination with cultural values [8]. Most of the respondents worked as employees of private companies (28.9%), public servants (28.5%), entrepreneurs (27.2%), and independent workers (8.5%).

Table 2 shows the distribution of respondents in this survey, with the middle class as the largest group. This does not proportionally represent people by class in the selected cities. Based on official statistical data, the upper social class in urban Indonesia still makes up less than 20% of the population.

## 2 Experimental design, materials, and method

This survey elaborates the cultural dimensions of consumerism, particularly focusing on the sub-dimensions of cultural values and cultural practice. Each sub-dimension is operationalized into a number of indicators to measure the workings of cultural aspects in the dynamics of food consumption at the household level [9]. These indicators are then developed in a questionnaire instrument. The following table operationalizes the concept of the cultural dimension:

All of the items in the cultural aspect in this dataset were measured using a five-point Likert scale. The questionnaire also included questions regarding respondents’ demographic characteristics and household income and expenditure and control questions about the importance of health factors and price factors in influencing food consumption.

Respondents to this survey were heads of household or their companions aged between 18 and 65 years in five major cities of Indonesia. These cities were chosen because they represent the major cities in Indonesia with diverse ethnic and religious heterogeneities within the scope of the urban context. In order to analyze social class, sampling in each city was conducted using a stratified sampling technique in the following stages: **first**, four sub-districts were randomly selected as the survey area; **second**, data were identified and a list of household populations was compiled for each sub-district; **third**, households were stratified based on their social class; **fourth**, proportional sampling of each household group was conducted based on their social class. The following table presents the number of survey samples and their proportions in each category of social class:

Data were collected through a survey of 710 respondents from five major cities in Indonesia: Jakarta with 174 samples, Bandung with 150 samples, Surabaya with 118 samples, Denpasar with 148 samples, and Makassar with 120 samples. The survey was conducted through face-to-face interviews, with which the research team was assisted by several local enumerators from local universities. The response rate of 82% indicates that the main samples in this survey were not all able to participate in data collection and an 18% reserve sample had to be used as substitutes.

Before the analysis was conducted, data were first processed through a process of coding, entering, and cleaning performed with the Statistical Package for Social Sciences (SPSS) program. The data were then processed by aggregation (computing) and re-categorization or recoding, especially for questions concerning the cultural dimension. The final step in processing data is to output data such as frequency tables and graphs. The data were analyzed using univariate analysis and cross table analysis. A number of questions related to demographic information were analyzed univariately by examining the largest percentage and the size of the concentration of the data, such as the mean. Cross-table analysis was mainly used on cultural aspects, which tend to be based on social class.

These survey data show the importance of the cultural dimension in understanding considerations concerning health, price, and cultural issues [10]. Cultural issues are shown as the most important factor in household food consumption, followed by health and price considerations [11], [12].

Table 7 shows the influence of social class on the factors of household food consumption [13], [14]. The outcome shows different numbers in every factor.

The cultural dimension is observed from the sub-dimensions of value and practice. Value is related to the influence of religious and traditional values on households’ food consumption patterns, while practice is related to practices or habits of food consumption patterns that are carried out in accordance with religious values or traditional values [4]. The survey data show that the sub-dimension of value (4.0) is more dominant in forming eating patterns than the sub-dimension of practice (3.6) [15].

Lastly, Table 8 presents the mean value of indicators based on value and practice categorization.

## CRediT authorship contribution statement

**Francisia SSE Seda:** Conceptualization, Methodology, Writing - original draft. **Lugina Setyawati:** Conceptualization, Methodology, Validation, Writing - original draft. **Timoti Tirta:** Validation, Data curation, Writing - original draft, Writing - review & editing. **Kevin Nobel:** Writing - review & editing.

## Appendix Supplementary materials

Image, application 1
