# Supplementary material for: Dataset on The Cultural Dimension of Urban Society Food Consumption in Indonesia
Source: Data Brief. 2020 May 13;31:105681. doi: 10.1016/j.dib.2020.105681 (PMC7251649; doi:10.1016/j.dib.2020.105681)
Supplement: Supplementary file 1 [file mmc1.docx]

| **QUESTIONNAIRE**  **The Cultural Aspects of Urban Society Food Consumption** |
| --- |

| **Respondent Code** |  |
| --- | --- |

**A. GENERAL INFORMATION**

| **Code** | **Questions** | **Category** |
| --- | --- | --- |
| 1. | City | 1) Jakarta 3) Surabaya 5) Denpasar  2) Bandung 4) Makasar |
| 2. | Province | 1) DKI Jakarta 3) East Java 5) Bali  2) West Java 4) South Sulawesi |

**B. DEMOGRAPHIC INFORMATION**

| **Code** | **Questions** | **Category** |
| --- | --- | --- |
| 3. | Gender | 1) Male 2) Female |
| 4. | Age |  |
| 5. | Religion | 1) Islam 3) Protestant 5) Budha  2) Catholic 4) Hindu 6) Other |
| 6. | Ethnic | 1. Java 3) Minang 5) Batak 7) Other   2) Sunda 4) Bali 6) Madura |
| 7. | The Highest Education Stage | 1. No. Education 6) Diploma 2. Primary School drop out 7) Bachelor degree 3. Primary school 8) Master degree 4. Junior High School 9) doctoral degree   5) Senior High School |
| 8. | How many years have you lived in urban areas? |  |
| 9. | Occupation | 1. No. Job 5) Teacher/Lecturer 2. Government Employees 6) Enterpreneur 3. Police officer/Army 7) Independent worker 4. Private Employees 8) Other |
| 10. | Social Class *(based on official data of income)* | 1. Lower Class 2) Middle Class 3) Upper Class |
| 11 | Number of household member |  |

**C. INCOME AND EXPENDITURE INFORMATION**

| **Code** | **Questions** | **Category** |
| --- | --- | --- |
| 12 | Total Household Income/monthly (in IDR) |  |
| 13 | Time of Income Receipt | 1) Daily 3) Monthly  2) Weekly |
| 14 | Total Household Expenditure/monthly (in IDR) |  |
| 15 | % monthly household expenditure FOR FOOD |  |

**D. CONTROL QUESTIONS**

**Notes : 1) SD: Strongly Disagree; 2) D: Disagree; 3) N: Neither agree nor disagree;**

**4) A : Agree ; 5) Strongly Agree**

| **Code** | **Statement** | 1 | 2 | 3 | 4 | 5 |
| --- | --- | --- | --- | --- | --- | --- |
| 16 | The Principle of Health is a major consideration in your family’s consumption patterns | SD | D | N | A | SA |
| 17 | The issue of Price is the main consideration in your family's consumption patterns | SD | D | N | A | SA |

**E. CULTURAL ASPECT OF FOOD CONSUMPTION**

**Notes : 1) SD: Strongly Disagree; 2) D: Disagree; 3) N: Neither agree nor disagree;**

**4) A : Agree ; 5) Strongly Agree**

| **Code** | **Statement** | 1 | 2 | 3 | 4 | 5 |
| --- | --- | --- | --- | --- | --- | --- |
| 18 | The principle of tradition/customs becomes the main consideration in choosing the type of food for your family's consumption | SD | D | N | A | SA |
| 19 | The principle of tradition/customs becomes the main consideration in choosing the place to eat together with your family | SD | D | N | A | SA |
| 20 | The principle of tradition/customs becomes the main consideration in consumption pattern of your family | SD | D | N | A | SA |
| 21 | The principle/religious value becomes the main consideration in choosing the type of food for your family consumption | SD | D | N | A | SA |
| 22 | The principle/religious value becomes the main consideration in choosing the place to eat together with your family | SD | D | N | A | SA |
| 23 | The principle/religious value becomes the main consideration in consumption pattern of your family | SD | D | N | A | SA |
| 24 | You always consume food products recommended by religious principle | SD | D | N | A | SA |
| 25 | You always consume custom-recommended food products | SD | D | N | A | SA |
| 26 | Retail / halal guaranteed place of sale (ahad mart and others) becomes your preference in buying food products | SD | D | N | A | SA |
| 27 | Type of dishes variations are important are important in your family's daily consumption menu according to your religious values | SD | D | N | A | SA |
| 28 | Typical dishes from the area of ​​origin husband/wife become part of the menu served For household consumption | SD | D | N | A | SA |
